# Diagnostic performance of metagenomic next-generation sequencing in *Mycobacterium* and non-*Mycobacterium* infection patients: a retrospective study at a large comprehensive medical center

**DOI:** 10.3389/fcimb.2026.1935536

**Published:** 2026-10-29

**Authors:** Xiaoli Zhou, Shengkun Peng, Rui Tan, Jiaqiang Wang, Honghua Hu, Kai Zhang, Aibo Liu

**Affiliations:** 1 Department of Laboratory Medicine, Sichuan Academy of Medical Sciences and Sichuan Provincial People’s Hospital, School of Medicine, University of Electronic Science and Technology of China, Chengdu, Sichuan, China; 2 Genetic Diseases Key Laboratory of Sichuan Province, Sichuan Clinical Research Center for Laboratory Medicine, Key Laboratory of Experimental Diagnostic Medicine designated by Health Commission of Sichuan Province, Chengdu, China; 3 Department of Radiology, Sichuan Provincial People’s Hospital, University of Electronic Science and Technology of China, Chengdu, China; 4 Department of Laboratory Medicine, West China School of Medicine, Sichuan University, Sichuan University affiliated Chengdu Second People’s Hospital, Chengdu Second People’s Hospital, Chengdu, Sichuan, China

**Keywords:** metagenomic next-generation Sequencing (mNGS), tuberculosis, NTM, cerebral spinal fluid, bronchoalveolar lavage fluid, hydrothorax and ascites, intrapulmonary, extrapulmonary

## Abstract

**Background:**

Metagenomic next-generation sequencing (mNGS) has become a potentially valuable complementary test, if not an essential one, in some challenging clinical cases. This study comprehensively compared mNGS with other methods, including TB-IgG, TB-IGRA, TB-DNA, Xpert, culture, and AFS for the diagnosis of and differentiation between TB infection from NTM infection using multiple sample types, including BALF, CSF, blood, pus, tissue, hydrothorax, and ascites.

**Methods:**

From 2021 to 2024, the clinical data, including imaging, laboratory results, and medical records, of 425 patients with suspected TB infection were retrospectively collected and classified as PTB [306 (72%)], EPTB [67 (15.76%)], and non-TB [52 (12.24%)], according to the final clinical diagnosis.

**Results:**

Among the three groups, significant differences were observed in age, sex, cough, smoking history, hsCRP, IFN-γ, and hypertension (*P* < 0.05). According to the final clinical diagnosis, the sensitivity and specificity of mNGS in all samples were 90.1% (95% CI: 0.866–0.927) and 75.0% (95% CI: 0.618–0.848), respectively. The sensitivity of mNGS was higher than that of TB-IGRA (79.1%), Xpert (72.4%), culture (52.3%), TB-DNA (45.6%), TB-IgG (36.4%), and AFS (10.7%). For distinguishing TB from non-TB, the sensitivity of mNGS was approximately 37.8% higher than that of standard test culture (90.1% vs 36.4%; *P* < 0.001). Further analysis also showed that MTB read counts from mNGS were highly correlated with MTB burden measured by other laboratory tests (*P* < 0.05). Analysis of the ROC curve of mNGS reads showed that the AUC for MTB diagnosis was 0.9073, with a sensitivity of 73.19% and specificity of 96.15% when the recommended cutoff value of 4 reads (Youden’s index, 0.6934) was applied.

**Conclusions:**

For patients with suspected TB or NTM infection, mNGS may serve as a helpful adjunctive tool, particularly when routine diagnostic tests are unrevealing or indeterminate. It has the potential to assist in distinguishing MTB from NTM and to provide additional evidence to guide appropriate treatment choices, though its results should always be interpreted alongside clinical findings and conventional microbiological testing.

## 1 Introduction

Tuberculosis (TB) is an infectious disease caused by bacteria of the *Mycobacterium tuberculosis* complex (MTBC), mostly affecting the lungs in approximately 70%–80% of cases (Lyon and Rossman, 2017). It can be transmitted through the air when the infected individuals cough, sneeze, or spit (Bagcchi, 2023; Churchyard et al., 2017). MTBC includes *M. tuberculosis, M. bovis, M. microti*, and *M. africanum*, and over 90% of tuberculosis infections in humans are caused by *M. tuberculosis* (Forrellad et al., 2013). TB infection can lead to not only active TB (disease status) but also latent TB infection. Typical TB symptoms include prolonged cough (sometimes with blood), fever, fatigue, night sweats, decreased appetite, and weight loss, which are similar to non-tuberculous mycobacteria (NTM) infection, including cough, fever, and weight loss (Lyon and Rossman, 2017; Zhang et al., 2024; Zhou et al., 2023). According to the World Health Organization (WHO), the TB incidence rate (new cases per 100,000 population per year) increased by an estimated 3.6% between 2020 and 2021, with approximately 10.6 million people developing TB worldwide in 2021, of whom China accounted for more than two-thirds (7.4%) of global TB cases (Bagcchi, 2023). Since 2019, TB has been the top cause of death from a single infectious agent and the 13th leading cause of death worldwide. In 2020 and 2021, however, TB ranked second as a cause of death from a single infectious agent, after COVID-19. Approximately 1.6 million people died from TB in 2021. Furthermore, the death rate was high among individuals with HIV infection or drug-resistant TB. Therefore, accurate and early detection of the pathogen is important for controlling person-to-person transmission, preventing the emergence of drug-resistant tuberculosis, and enabling physicians to initiate prompt treatment.

The etiological agent *Mycobacterium tuberculosis* (MTB), which has a single, circular chromosome of approximately 4.4 Mb, can avoid host cell clearance by arresting the normal macrophage maturation process; as a result, it can cause not only pulmonary tuberculosis (PTB) but also extrapulmonary tuberculosis (EPTB) (Koch and Mizrahi, 2018). MTB infection can cause pulmonary involvement, which is common and affects more than 75% of the total number of cases. In addition, MTB can invade extrapulmonary organs, including the brain (Thwaites et al., 2013), kidneys (Suzuki and Morisawa, 2022), intestines (Kentley et al., 2017), and bones and joints (Hogan et al., 2019). MTB invades macrophages and replicates in large quantities, mainly by utilizing multiple pattern recognition receptors (PRRs) on the host surface, such as Toll-like receptors (TLRs) and nucleotide-binding oligomerization domain-like receptors (NOD-like receptors, NLRs) (Kleinnijenhuis et al., 2011). The successful infection process of MTB is very complex, mainly including the following: 1) successful replication in macrophages; 2) modification of the host immune response, allowing the host to control the infection process but not eradicate the bacteria; and 3) the ability to remain relatively inactive in the host while retaining the potential for reactivation (Koch and Mizrahi, 2018; Pai et al., 2016). Based on the pathogenesis, MTB infection can be divided into latent tuberculosis infection (LTBI) and active tuberculosis (ATB). The outcome of different infection states is closely related to the amount and virulence of MTB, in addition to the host’s immune function (Pai et al., 2016). *Mycobacterium tuberculosis* grows slowly, with a generation time of approximately 20 h in laboratory media. An estimated one-fourth of the world’s population has latent *M. tuberculosis* infection, which can persist for decades before reactivating to cause post-primary tuberculosis in humans (Koch and Mizrahi, 2018). Drug resistance is mediated exclusively by chromosomal mutations and rearrangements. Besides, it is difficult to distinguish MTB from NTM based on imaging and laboratory results in some cases. Tuberculosis is preventable and curable, so early detection of TB, initiation of effective treatment to interrupt transmission, and screening and treatment of patients with LTBI who are at high risk of progressing to active TB might be practical strategies for reducing the TB epidemic in China (Liu et al., 2017).

To date, in clinical laboratories, routine tests for *Mycobacterium tuberculosis* detection include: 1) immunological testing methods: TB interferon-γ release assay (TB-IGRA), *Mycobacterium tuberculosis* IgG antibody (TB-IgG), and purified-protein derivative (PPD) skin test (TST); 2) molecular testing methods: MTB DNA detection (TB-DNA) and GeneXpert MTB/RIF assay (hereinafter referred to as “Xpert”); and 3) traditional microbiological methods: MTB culture and acid-fast bacilli staining (AFS). However, the sensitivity and specificity of these routine detection methods in the diagnosis of tuberculosis are limited. TB-DNA and Xpert have not substantially improved global case detection rates and have shown limited efficacy in extrapulmonary TB (2013). Besides, TB-IGRA, TB-IgG, and TST can be influenced by the host’s immune response status, which can substantially affect the sensitivity and specificity of the test results (Hang et al., 2011; Lv et al., 2020; Shi et al., 2018; Xu et al., 2018). In addition, TST can cause greater damage to the human body, and there are certain risks. MTB isolation and culture test are considered the gold standard method for tuberculosis diagnosis, but it takes a long time from testing to diagnosis and therefore of limited value for early and rapid diagnosis (Rasool et al., 2019). Hence, early and accurate detection of *Mycobacterium tuberculosis* is crucial for the diagnosis of tuberculosis infection, including both pulmonary and extrapulmonary tuberculosis.

Nowadays, a new technology, metagenomics next-generation sequencing (mNGS), has emerged for the diagnosis of clinical infectious diseases since its first use in 2008. The mNGS method, which provides broad coverage and rapid turnaround, enables non-targeted, rapid, and accurate auxiliary diagnosis of pathogens, including bacteria, viruses, fungi, and parasites, and has been widely used in the accurate diagnosis of clinical infectious diseases, including respiratory tract infections, circulatory system infections, urinary tract infections, gastrointestinal tract infections, musculoskeletal system infections, and central nervous system infections (Li et al., 2018, 2021; Piantadosi et al., 2021; Stratton et al., 2021; Tao et al., 2022; Wilson et al., 2014, 2019; Zhan et al., 2021; Zhou et al., 2022). Sample types include, but are not limited to, blood, tissue, bronchoalveolar lavage fluid (BALF), sputum, cerebrospinal fluid (CSF), hydrothorax, and ascites. Thus, combined with routine tests, mNGS can not only help physicians diagnose and treat patients as soon as possible but also help patients improve prognosis, especially when other tests are false negative because of immunodeficiency status of the host or other factors.

However, most previous studies have focused primarily on case reports of mNGS applications, and few have systematically evaluated its diagnostic performance in pulmonary TB, extrapulmonary TB, and NTM infections. Therefore, we conducted a single-center prospective clinical study in Sichuan, China, to investigate the potential auxiliary diagnostic role of mNGS for detection of MTB and NTM pathogens in different clinical sample types from human patients, compared with routine diagnostic methods, including TB-IgG, TB-IGRA, TB-DNA, Xpert, culture, and AFS, and clinical imaging and final diagnosis.

## 2 Methods

### 2.1 Study design and criteria

This retrospective observational study included inpatients who underwent mNGS testing at our hospital between 2021 and 2024. Eligible patients were selected from the hospital information system, and their medical history, imaging examinations, and laboratory test results were recorded and evaluated. The diagnosis was established according to the Chinese Clinical Treatment Guide for Tuberculosis (2017 Edition) and WHO recommendations, based on microbiological evidence (positive culture or Xpert MTB/RIF), radiological findings (chest CT), clinical symptoms (cough ≥ 2 weeks, fever, night sweats, and weight loss), clinical response to anti-TB treatment, and exclusion of other possible diagnoses. The inclusion criteria were: (1) patients who were clinically suspected of having tuberculosis infection and presented with related symptoms, such as fever, night sweats, and weight loss; (2) patients whose imaging results were consistent with the diagnosis; and (3) patients who underwent mNGS testing, with or without additional tuberculosis-related tests, such as TB-IgG, TB-IGRA, TB-DNA, culture, Xpert, or AFS. The exclusion criteria were (1) no mNGS test and (2) incomplete medical history.

The criteria for the diagnosis of PTB and EPTB were based on the WHO diagnostic guidelines and the Chinese Clinical Treatment Guide for Tuberculosis (2017 Edition). PTB refers to any bacteriologically confirmed or clinically diagnosed case of TB involving the lung parenchyma or tracheobronchial tree. EPTB refers to any bacteriologically confirmed or clinically diagnosed case of TB involving organs other than the lungs (e.g., meninges, bones and joints, and abdomen). Otherwise, patients were classified as non-TB cases, including infectious diseases caused by other pathogens, such as NTM, or non-infectious diseases, such as tumors and autoimmune diseases. The imaging films were re-examined by one radiologist who was unaware of the patients’ clinical data. Imaging examinations were retrospectively reviewed and interpreted for possible lesions. This research was approved by the Medical Professional Committee of the Sichuan Provincial People’s Hospital (Permit Number: 2022172), with a waiver of informed consent because of the retrospective nature of the study.

### 2.2 Sample collection

A lumbar puncture was performed to obtain cerebrospinal fluid (CSF) according to standard procedures. Tissue samples were collected without formalin. Whole blood, BALF, sputum, hydrothorax, ascites, pus, and other suspected lesions were collected according to standard procedures.

### 2.3 TB-IgG

The test procedure and interpretation of the results were performed in strict accordance with the instructions of the TB IgG antibody detection kit (enzyme-linked immunosorbent assay) (Wantai Biology Co., Ltd, Beijing, China), which is used to detect TB-IgG antibodies in human serum or plasma samples. TB-IgG antibodies may persist for a long time during the course of tuberculosis and can be used as an auxiliary diagnostic marker of tuberculosis infection.

### 2.4 TB-IGRA

The test was used to qualitatively detect the specific T-cell immune response to *Mycobacterium tuberculosis* in fresh anticoagulated peripheral venous blood samples *in vitro*. It was performed strictly following the manufacturer’s instructions (Wantai Kairui Biology Co., Ltd, Xiamen, China) (Qiu et al., 2015). Briefly, heparinized whole blood, which was inverted and mixed more than three times, was collected from each patient and inoculated into three tubes of 1 mL within 16 h: one containing specific MTB antigens (ESAT-6 and CFP-10), a positive control tube containing phytohemagglutinin-P, and a negative control tube containing no antigens. Then samples were incubated for 20–24 h at 37 ˚C, with the culture tubes kept upright during the cultivation process. Next, after centrifugation at 3,000–5,000 rpm for 10 min, plasma samples were collected for IFN-γ quantification using a direct acridinium ester-based double-antibody sandwich chemiluminescence immunoassay. The result was considered positive if the IFN-γ level after stimulation with TB antigens minus the negative control was ≥14 pg/mL and ≥25% of the negative control. The test was considered negative if the IFN-γ level was <14 pg/mL (after subtraction of the negative control). The test result was considered indeterminate if the positive control minus negative control was <20 pg/mL. The linear range of detection was 1 pg/mL to 5,000 pg/mL.

### 2.5 TB-DNA

TB-DNA was identified using a PCR fluorescent probe technique with a nucleic acid detection kit that incorporated both positive and negative controls (DaAn Gene, Guangzhou, China). The test procedure and interpretation of results were performed according to the manufacturer’s guidelines. The minimum detectable limit was 500 copies/mL.

### 2.6 Xpert

Specimens included BALF, urine, tissue, pleural fluid, ascites fluid, CSF, and other clinical specimens. For sputum, Xpert sample processing reagent (SR) containing NaOH and isopropanol was added to the sample container at a ratio of 2:1, vortexed for at least 10 s, and then incubated for 15 min at room temperature. Other liquid specimens, including BALF, CSF, urine, and pleural fluid, were first centrifuged in a 15-mL centrifuge tube at 3,000 g for 15 min. The supernatant was then discarded, and 2–4 mL of SR was added and mixed thoroughly with the precipitate. The samples were assessed by Xpert (Cepheid AB, Solna, Sweden) according to the manufacturer’s instructions. Finally, we transferred the mixture into an Xpert cylinder and loaded it into a GeneXpert Dx instrument to record the results.

### 2.7 Culture

Mycobacterium culture included the BD BACTEC™ MGIT™ 960 system, a fully automatic mycobacterial detection system, and Lowenstein Jensen (L-J) solid culture medium. The procedures were performed according to the manufacturer’s instructions. Specimens were decontaminated with an equal volume of 2% N-acetyl-L-cysteine-sodium hydroxide solution, vortexed vigorously for 20 s, and then incubated for 15–20 min at room temperature (not exceeding 20 min to prevent killing of *Mycobacterium tuberculosis*). Sterile phosphate buffer (pH 6.8) was then added, followed by centrifugation at 3,000 g for 15 min. The sediment was resuspended in 1–3 mL of phosphate buffer (pH 6.8). Then, 500 µL of concentrated specimen was inoculated into an MGIT tube containing 800 uL of mycobacterial growth indicator tube (MGIT), and the culture was performed using the MGIT 960 system (Becton, Dickinson and Company, USA) according to the manufacturer’s instructions and protocols. Simultaneously, 100 µL of concentrated specimen was inoculated into Lowenstein Jensen (L-J) solid culture medium (BaSO, Zhuhai, China) and incubated at 35 °C–37 °C for 42 days. MGIT™ tubes were then incubated for 42 days, with continuous sample monitoring until they were automatically identified as positive by MGIT™ equipment. Positive tubes or cultures were observed under a microscope.

### 2.8 AFS

For aseptic body fluid samples, such as urine, pus, ascites fluid, pleural fluid, BALF, and CSF, 2–4 mL of sample was added to a 15-mL sterile disposable centrifuge tube. The supernatant was discarded after centrifugation at 3,000 rpm for 15 min, and the precipitate was used for acid-fast bacillus staining (BA4091, BaSO, Zhuhai, China) and smear microscopy (OLYMPUS, Japan).

### 2.9 mNGS sample processing and DNA extraction

For CSF, BALF, sputum, pleural fluid, tissue, and ascites fluid, samples (600 μL each) were collected from patients according to the standard protocol.

1. Wall-breaking reaction: lysozyme (7.2 μL) was added to the 600 μL sample in a new 1.5 mL centrifuge tube and incubated in a metal bath at 30 °C. Then the mixture was transferred to a new 2-mL centrifuge tube containing 250 μL 0.5-mm glass beads on a wall breaker and homogenized under the following conditions: 6 m/s, 45 s, two cycles, and a 2-min interval.
2. The mixture was then briefly centrifuged for later use.
3. DNA extraction: 0.3mL of the above mixture was transferred into a new 1.5 mL centrifuge tube for DNA extraction using the TIANamp Micro DNA Kit (DP316, TIANGEN BIOTECH) according to the manufacturer’s protocol.

The extracted DNA was used to construct DNA libraries. Because blood contains free DNA and RNA, DNA extraction from blood began at step 3.

### 2.10 Construction of mNGS libraries and sequencing

The NEBNext Ultra II DNA Library Prep Kit (New England Biolabs Inc.) was used to construct Illumina sequencing libraries, and a Nextseq 550 DX (75 bp single-end reads; Illumina) was used for sequencing. Approximately 20 million reads were generated for each sample.

### 2.11 mNGS bioinformatic analysis

High-quality sequencing data were generated by removing low-quality reads, including adapter contamination, duplicated reads, and short reads (Illumina: length < 50 bp), followed by computational subtraction of human host sequences mapped to the human reference genome (GRCh38) using the Burrows–Wheeler Alignment, and removal of low-complexity reads. The remaining sequencing data were aligned to the NCBI database using SNAP and processed for advanced data analysis, including taxonomic annotation, genome coverage/depth calculation, and abundance calculation.

### 2.12 Statistical analysis

The sensitivity, specificity, positive predictive value, negative predictive value, and Youden index of mNGS, TB-IgG, TB-IGRA, TB-DNA, Xpert, culture, and AFS were calculated. Parallel and serial diagnostic tests were also conducted. McNemar’s test and Cohen’s kappa test for a paired fourfold table were performed to compare the results of mNGS and the traditional gold-standard methods: culture or Xpert. A non-parametric Mann–Whitney U test was used for comparisons between groups. All statistical analyses were performed using GraphPad Prism (version 6.01, GraphPad Software Inc.), SPSS (Version 25, IBM Corp.), and R (version 4.1.2). Continuous variables were expressed as mean ± standard deviation (SD). Categorical variables were represented as frequencies or percentages (%). A two-tailed *P* value < 0.05 was considered statistically significant. Subsequently, heat maps were constructed using the R package pheatmap to visualize the distribution of pathogens. Receiver operating characteristic (ROC) curves were generated, and the area under each curve (AUC) was calculated.

## 3 Results

### 3.1 Characteristics of the study population

Samples from 4,150 patients underwent mNGS testing at Sichuan Provincial People’s Hospital between 2021 and 2024, and 425 patients who met the inclusion criteria were enrolled in this study (**Figure 1**). Of these, patients were divided into three groups based on diagnostic methods: PTB group (n = 306), EPTB group (n = 67), and non-TB group (n = 52). The demographic and clinical characteristics of these individuals are shown in **Table 1**. There were significant differences in age, sex, cough, smoking history, hsCRP, IFN-γ, and hypertension among the three groups (*P* < 0.05, respectively).

**Figure 1:**
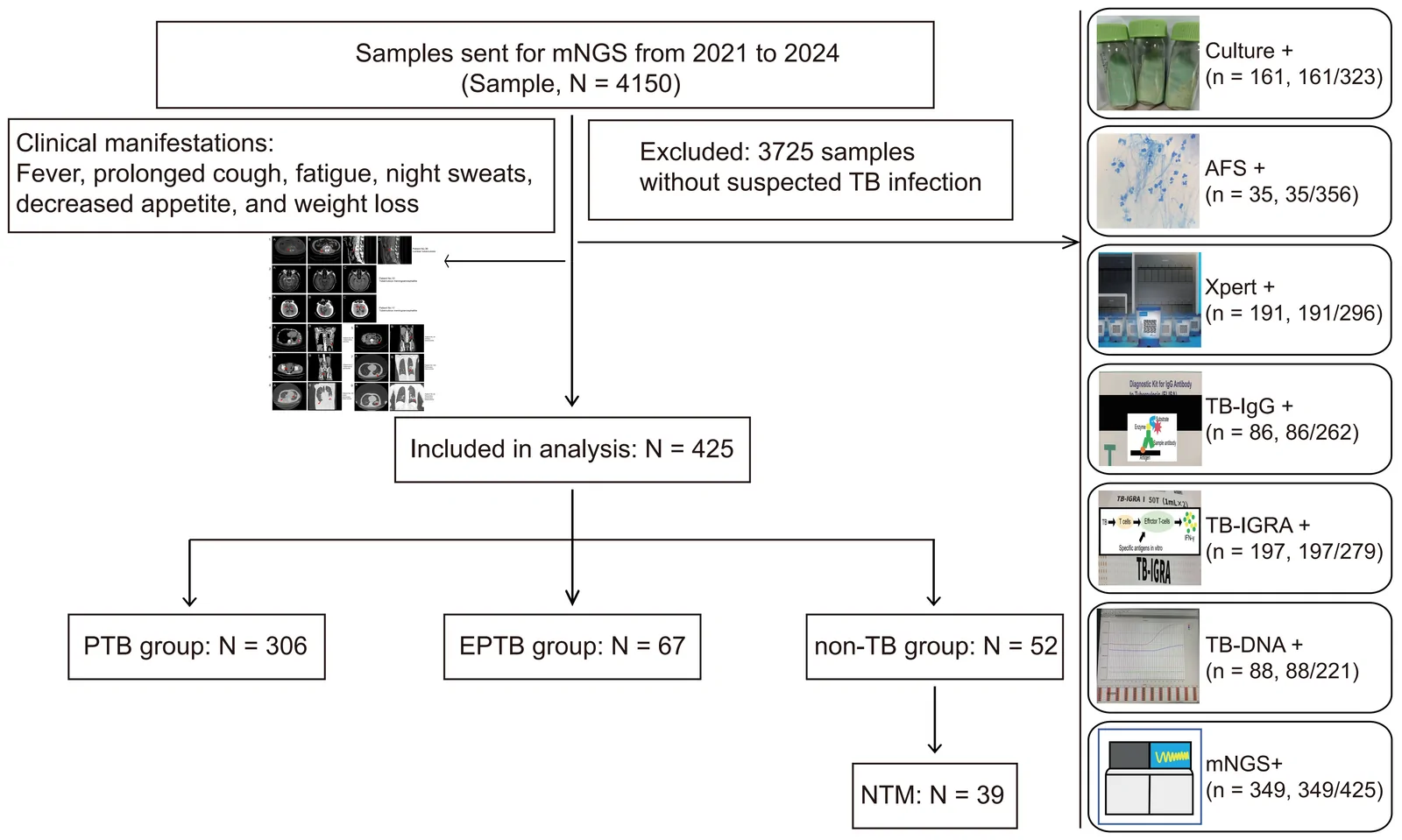
Flow diagram of the patients enrolled in this retrospective clinical study.

**Table 1:** General demographic and clinical characteristics of all enrolled patients in three groups.

Clinical characteristics	PTB group (n=306)	EPTB group (n=67)	Non-TB group (n=52)	*P*
Median age, years; Age, mean ± SD (years)	56.29 ± 19.43	48.67 ± 17.87	62.5 ± 16.88	0.0003*
Gender, n (%)				
Female	111 (36.27)	35 (52.24)	26 (50.00)	0.0177*
Male	195 (63.73)	32 (47.76)	26 (50.00)	
Symptoms, n(%)				
Cough	179 (58.50)	29 (43.28)	2 (3.85%)	<0.0001*
Fever	100 (32.68)	15 (22.39)	14 (26.92)	0.2151
Fatigue	35 (11.44)	3 (4.48)	6 (11.54)	0.2290
Night sweats	10 (3.27)	0 (0)	0 (0)	–
Smoking history, n (%)				
Smoker	53 (18.21)	1 (1.64)	3 (5.77)	0.0005*
Nonsmoker	238 (81.79)	60 (98.36)	49 (94.23)	
Pulmonary samples, n (%)				
BALF	264 (86.27)	1 (1.49)	42 (80.77)	<0.0001*
Sputum	1 (0.33)	–	–	–
Lung tissue	5 (1.63)	–	–	–
Extrapulmonary samples, n (%)				
CSF	13 (4.25)	21 (31.34)	1 (1.92)	<0.0001*
Pleural fluid	3 (0.98)	3 (4.48)	–	0.0739
Ascites	–	2 (2.99)	1 (1.92)	–
Pus	–	2 (2.99)	–	–
Secretion	–	1 (1.49)	–	–
Urine	–	1 (1.49)	–	–
Blood	18 (5.88)	5 (7.46)	6 (11.54)	0.3195
Bone marrow	2 (0.65)	1 (1.49)	1 (1.92)	0.5989
Joint fluid	–	3 (4.48)	1 (1.92)	–
Tissue	–	27 (40.30)	–	–
Laboratory tests positive, n (%)				
TB-IgG positive	73 (23.86)	10 (14.93)	3 (5.77)	0.0046*
TB-IGRA positive	171 (55.88)	22 (32.84)	4 (7.69)	<0.0001*
TB-DNA positive	82 (26.80)	6 (8.96)	0 (0)	<0.0001*
Xpert positive	176 (57.52)	15 (22.39)	0 (0)	<0.0001*
Culture positive	131 (42.81)	15 (22.39)	15 (28.85)	0.561
AFS positive	32 (10.46)	1 (1.49)	2 (3.85)	0.0925
mNGS TB positive	277 (90.52)	59 (88.06)	13 (25.00)	<0.0001*
mNGS NTM positive, n	1	0	39	–
Previous history of tuberculosis, n	33	7	7	–
Comorbidity, n (%)				
Diabetes	29 (9.97)	1 (1.64)	5 (9.62)	0.1066
Tumors	6 (2.06)	0 (0)	0 (0)	–
Hypertension	55 (18.9)	10 (16.39)	18 (34.62)	0.0242*
Laboratory examination, mean ± SD				
PCT (ng/mL)	3.23 ± 17.01	1.76 ± 7.17	1.13 ± 2.52	0.6294
hsCRP (mg/dL)	66.75 ± 66.72	35.65 ± 40.47	61.34 ± 68.61	0.0023*
Lymphocytes (x10^9^/L)	1.02 ± 0.78	0.89 ± 0.61	1.02 ± 0.72	0.4418
Lymphocytes percentage (%)	16.76 ± 11.77	15.26 ± 11.34	14.26 ± 12.58	0.0964
IFN-γ (pg/mL)	567.15 ± 1487.51	373.72 ± 646.18	15.73 ± 41.16	<0.0001*
ESR (mm/h)	64.86 ± 38.00	53.02 ± 33.77	65.27 ± 36.56	0.1538
CSF routine(10^6^/L)				
CSF white cell count	99.33 ± 128.38	179 ± 141.94	330	0.0725
CSF lymphocytes	60.86 ± 96.80	125.06 ± 122.48	318.45	0.0487*
CSF biochemistry				
CSF protein (g/L)	1.09 ± 0.94	2.25 ± 2.68	1.47	0.0506
CSF glucose (mmol/L)	2.77 ± 1.22	1.97 ± 1.36	5.48	0.0517
CSF Cl (mmol/L)	117.78 ± 6.37	111.74 ± 8.78	124.1	0.0595

BALF, bronchoalveolar lavage fluid; CSF, cerebrospinal fluid; TB, tuberculosis; AFS, acid-fast staining; PCT, procalcitonin; hsCRP, high sensitive C-reactive protein; SD, standard deviation.

'*' indicate statistical significance.

Among the 306 PTB patients, the median age was 56 (3–91) years, 18.21% (n = 53) were smokers (*P =* 0.0005), and most were men (63.73%, n = 195; *P* = 0.0177). The most common comorbidity was hypertension (18.9%, n = 55; *P* = 0.0242). The most frequent sample type was BALF (n = 264), with a significant difference among the three groups (*P* < 0.0001). The number of patients diagnosed with PTB for the first time was 273, whereas 33 had a history of TB and had previously received anti-tuberculosis treatment. In the EPTB group, the median age was 49 (6–80) years. The most common comorbidity was hypertension (16.39%, n = 10). A total of 60 patients were diagnosed with EPTB for the first time, whereas 7 had a history of TB and had previously received anti-tuberculosis treatment. The top three sample types in the EPTB group were tissues, CSF, and blood. The extrapulmonary TB cases included tuberculosis meningitis/meningoencephalitis (n = 24), tuberculous pleurisy (n = 4), genitourinary TB (n = 1), osteoarticular/spinal/joint tuberculosis (n = 13), tuberculous peritonitis (n = 3), lumbar tuberculosis (n = 17), skin tuberculosis (n = 2), and lymphadenitis TB (n = 1) (**Supplementary Table 1**). The median CSF white blood cell count was 179 (<2.5–589) ×10^6^/L, CSF lymphocyte count was 125.06 (7.1–527.744) ×10^6^/L, CSF protein was 2.25 (0.38–12.2) × g/L, CSF glucose was 1.97 (0.4–6.03) × mmol/L, and CSF Cl was 111.74 (96.8–126.4) × mmol/L. Only CSF lymphocyte count showed a significant difference among the three groups (*P* = 0.0487). In addition, among the 52 non-TB patients, 39 cases were diagnosed with NTM infection. A total of 13 cases were diagnosed with other diseases despite having low-read-count mNGS-positive results; two of these patients had a history of TB infection.

### 3.2 Overall manifestation of mNGS and clinical laboratory tests

In this study, multiple clinical laboratory tests, including TB-IgG, TB-IGRA, TB-DNA, Xpert, culture, and AFS, along with mNGS, were performed for TB detection. Because this was a retrospective clinical study, results for TB-IgG, TB-IGRA, TB-DNA, Xpert, culture, and AFS were undetermined for 262, 279, 221, 296, 323, and 356 patients, respectively (**Table 2**). In the PTB and EPTB groups (n = 373), patients were defined as having TB involving various organs according to either positive clinical laboratory tests results or comprehensive clinical evidence. The results of TB-IgG, TB-IGRA, TB-DNA, Xpert, culture, AFS, and mNGS for MTBC detection are summarized in **Figure 2**. In comparison, the Xpert assay demonstrated the highest positive detection rate (57.52%) among all clinical laboratory tests (TB-IgG 23.86%, TB-IGRA 55.88%, TB-DNA 26.8%, culture 42.81%, AFS 10.46%) in the PTB group, which was substantially lower than that of mNGS (90.52%; **Figure 2** and **Table 1**). In the EPTB group, TB-IGRA showed the highest positive detection rate (32.84%) among all other clinical laboratory tests (TB-IgG 14.93%, TB-DNA 8.96%, Xpert 22.39%, culture 22.39%, AFS 1.49%), which was substantially lower than that of mNGS (88.06%; **Figure 2** and **Table 1**). Remarkably, 13 positive TB-detection events were observed in the non-TB group (n = 52) using mNGS, although the final diagnoses were based on clinical manifestations. However, the positive rate of culture reached 28.85%, which was higher than those of the other approaches (TB-IgG 5.77%, TB-IGRA 7.69%, AFS 3.85%), and the positive results from these assays were mostly indicative of NTM infection. Among the 373 patients confirmed as TB-positive, we analyzed all the tests actually performed on each patient from a panel of seven TB-detection methods. Only four patients (1.07%) showed consistently positive results across all seven assays.

**Table 2:** Mycobacterium-detection performance comparison between mNGS and other methods.

Test method	PTB group (n=306)	EPTB group (n=67)	Non-TB group (n=52)
mNGS			
TB+	277	59	13
TB-	28	8	0
NTM+	1	0	39
NA	0	0	0
Xpert			
+	176	15	0
–	58	15	32
NA	72	37	20
Culture			
+	131	15	15
-	115	18	29
NA	60	34	8
AFB staining			
+	32	1	2
-	241	35	45
NA	33	31	5
TB-IgG			
+	73	10	3
-	123	22	31
NA	110	35	18
TB-IGRA			
+	171	22	4
-	46	5	31
NA	89	40	17
TB-DNA			
+	82	6	0
-	99	6	28
NA	125	55	24

**Figure 2:**
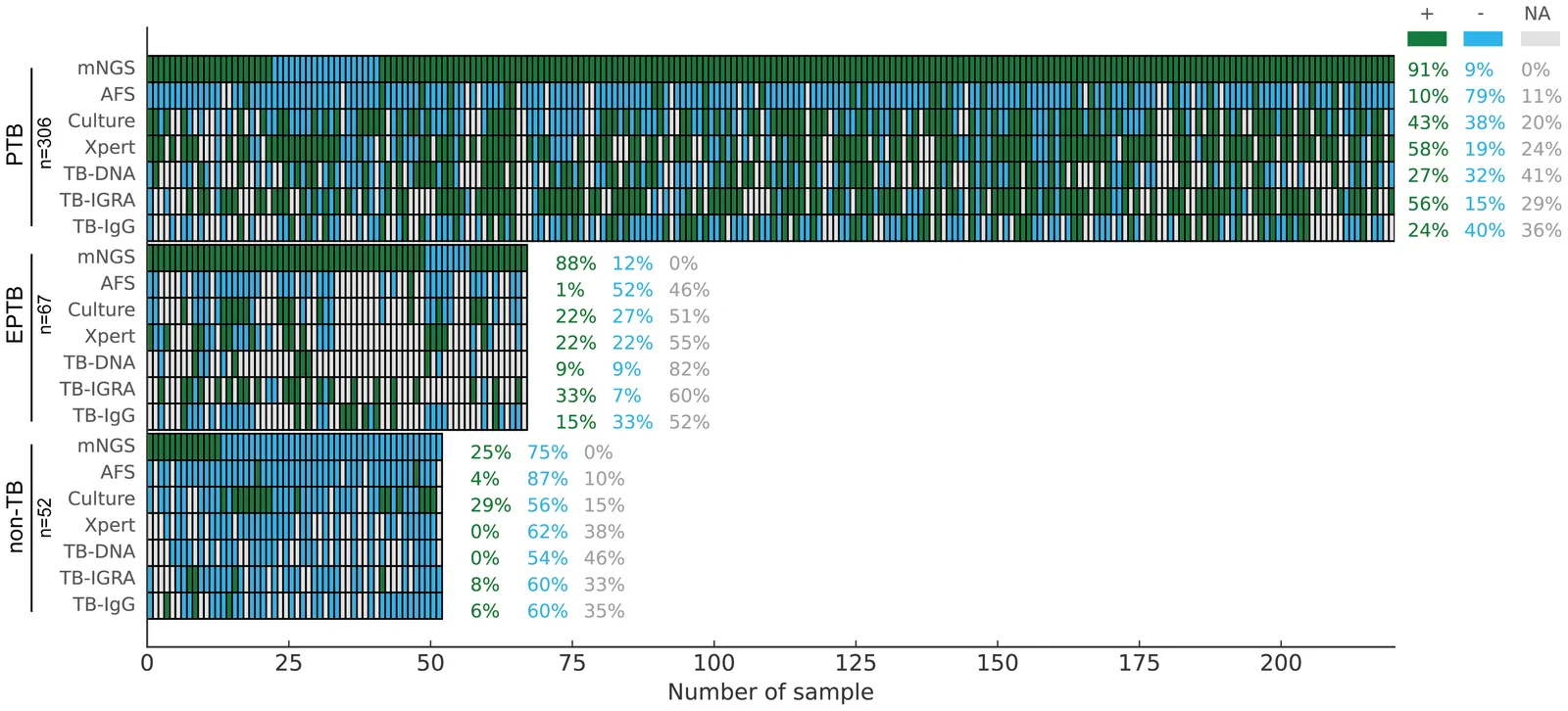
TB-detection results of TB-IgG, TB-IGRA, TB-DNA, Xpert, culture, AFS, and mNGS tests. Positive and negative TB detection by each method are indicated in green and blue, respectively. The top panel represents the clinically diagnosed PTB patients, the middle panel represents the clinically diagnosed EPTB patients, and the bottom panel represents the non-TB group. Positive and negative detection rates are shown on the right using a color scale.

In the PTB group, 91 patients underwent all seven assays (mNGS, TB-IgG, TB-IGRA, TB-DNA, Xpert, culture, and AFS). In contrast, only 2 patients in the EPTB group and 14 in the non-TB group completed all seven tests; these two groups were not further analyzed owing to the small sample sizes. Among the PTB patients who underwent all seven assays (n = 91), TB-IGRA demonstrated the highest positive detection rate (84.62%) among the conventional laboratory tests, followed by Xpert (78.02%), culture (61.54%), TB-DNA (45.05%), TB-IgG (39.56%), and AFS (14.29%), whereas mNGS achieved a positive rate of 100.00% (**Supplementary Table 2**). Compared with the overall PTB cohort (including patients with incomplete data), the complete-case subset (n = 91) showed higher positive rates across all seven assays. The rank order of positive rates among the seven assays was generally consistent between the two groups, except that TB-IGRA ranked above Xpert in the complete-case subset, whereas the opposite order was observed in the unselected cohort.

### 3.3 Detection results and concordance of mNGS and other six clinical routine tests

As shown in **Figure 3**, among the 425 enrolled patients, mNGS was the most effective method among the seven tests, detecting MTBC in 349/425 patients (82.12%; *P* < 0.05). However, TB-IGRA and Xpert showed similar overall MTBC detection efficiency (197/297, 70.61% vs 191/296, 64.53%; *P* > 0.05). Similarly, TB-DNA showed a similar overall MTBC detection rate to those of TB-IgG and MTBC culture (88/221, 39.82% vs 86/262, 32.82%, and 161/323, 49.85%, respectively; *P* > 0.05). Because culture and Xpert are well-established methods for MTBC detection, we focused on analyzing the correlation among these three methods. There were 270 patients whose results were tested by mNGS, Xpert, and culture at the same time. Among these 270 patients, the results of mNGS, Xpert, and culture matched completely in 116 (42.96%) patients, including 91 PTB and 5 EPTB patients with positive findings in all three methods. The detection results of the remaining 154 samples were conflicting, including 45 samples positive by mNGS only (16.67%), 10 samples positive by Xpert only (3.7%), 18 samples positive by culture only (6.67%), 60 samples positive by mNGS and Xpert (22.22%), 13 samples positive by mNGS and culture (4.81%), and 8 samples positive by Xpert and culture (2.96%) (**Figure 3B**). We also analyzed the detection efficiency of the seven methods in the 306 PTB and 67 EPTB patients (**Figures 3D, G**). Of the 306 PTB patients, 214 were tested using mNGS, Xpert, and culture. Among these 214 samples, 91 (91/214, 42.52%) were positive by mNGS, Xpert, and culture, 55 (55/214, 25.7%) were positive by mNGS and Xpert, 8 (8/214, 3.74%) were positive by mNGS and culture, and 34 (34/214, 15.89%) were positive by mNGS only (**Figures 3E, F**). Of the 67 EPTB patients, 24 were tested using mNGS, Xpert, and culture. Among these 24 samples, 5 (5/24, 20.83%) were positive by mNGS, Xpert, and culture, 5 (5/24, 20.83%) were positive by mNGS and Xpert, 5 (5/24, 20.83%) were positive by mNGS and culture, and 5 (5/24, 20.83%) were positive by mNGS only (**Figures 3H, I**).

**Figure 3:**
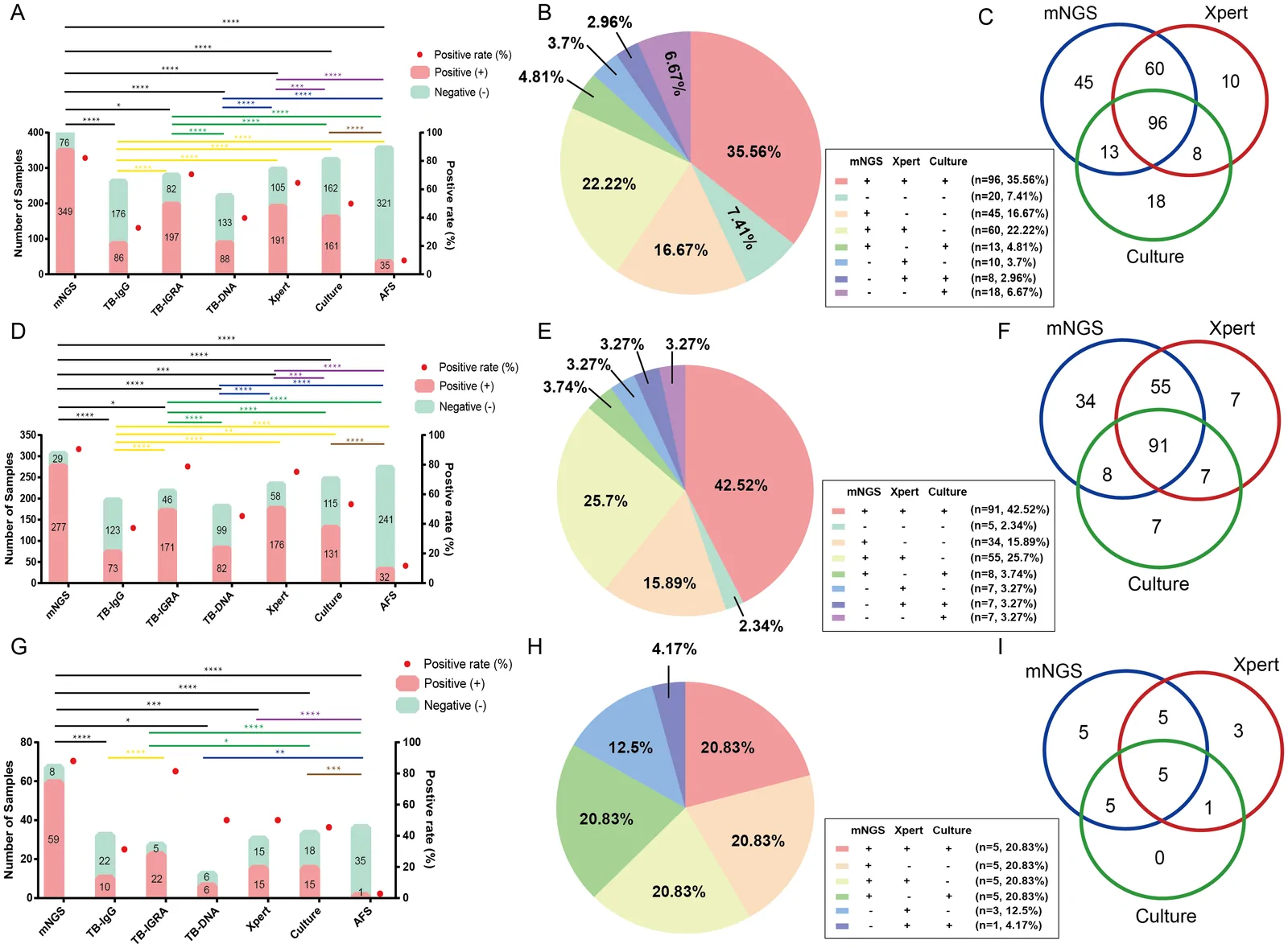
Comparison of mNGS, TB-IgG, TB-IGRA, TB-DNA, Xpert, culture, and AFS for the detection of MTBC. **(A)** The number of positive and negative findings for each of the seven methods in all 425 patients. **(B, C)** The number and percentage of patients with positive or negative findings by mNGS, Xpert, and culture methods in 270 patients are shown in the pie chart and Venn diagram. **(D)** The number of positive and negative findings for each of the seven methods in 306 PTB patients. **(E, F)** The number and percentage of patients with positive or negative findings by mNGS, Xpert, and culture methods in 214 patients are shown in the pie chart and Venn diagram. **(G)** The number of positive and negative findings for each of the seven methods in 67 EPTB patients. **(H, I)** The number and percentage of patients with positive or negative findings by mNGS, Xpert, and culture methods in 24 patients are shown in the pie chart and Venn diagram. *P < 0.05; **P < 0.01; ***P < 0.001, ****P < 0.0001..

Additionally, we carried out McNemar’s test and the consistency test based on the different classifications of clinical specimens to better understand the relationship between mNGS and culture or Xpert (**Table 3**). The correlation between mNGS and culture in all 425 specimens was statistically significant (*P* < 0.001). However, the reported kappa statistic was 0.267 (*P* = 0.605), indicating no concordance between the two methods. Similarly, McNemar's test revealed a significant relationship between mNGS and culture in the 313 pulmonary samples (P<0.001); however, the kappa statistic indicated poor concordance (κ = 0.080, *P* = 0.777) and 112 extrapulmonary samples (*P* = 0.001; kappa = 1.204, *P* = 0.273; **Table 4**). A significant relationship and correlation between mNGS and Xpert were also observed in all samples (*P* < 0.001; kappa = 33.480, *P* < 0.001), pulmonary samples (*P* < 0.001; kappa = 45.262, *P* < 0.001), and extrapulmonary samples (*P* = 0.043; kappa = 0.015, *P* = 0.901). The kappa statistic indicated satisfactory concordance between mNGS and Xpert in all and pulmonary samples, but not in extrapulmonary samples (**Table 3**).

**Table 3:** Relationship between mNGS and traditional golden methods culture and Xpert.

mNGS of samples	Culture		P1	P2	Xpert		P1	P2
	P+	N-			P+	N-		
All samples, n (%)								
Positive	129(80.12)	126(77.78)	<0.001*	0.605	173(90.58)	66(62.86)	<0.001*	<0.001*
Negative	32(19.88)	36(22.22)			18(9.42)	39(37.14)		
Pulmonary samples, n (%)								
Positive	109(80.74)	101(82.11)	<0.001*	0.777	151(94.38)	45(59.21)	<0.001*	<0.001*
Negative	26(19.26)	22(17.89)			9(5.62)	31(40.79)		
Extrapulmonary samples, n (%)								
Positive	20(76.92)	25(64.10)	0.001*	0.273	22(70.97)	21(72.41)	0.043	0.901
Negative	6(23.08)	14(35.90)			9(29.03)	8(27.59)		

Statistically significant data were marked with asterisk. mNGS, metagenomic next-generation sequencing; Xpert, GeneXpert MTB/RIF assay; P1, P value of McNemar’s test; P2, P value of Cohen’s Kappa test.

'*' indicate statistical significance.

**Table 4:** Diagnostic efficiency of mNGS and traditional methods in TB.

Samples	Sensitivity (95%CI)	Specificity (95%CI)	PPV (95%CI)	NPV (95%CI)	Youden Index
All samples					
TB-IgG	36.4% (0.302-0.430)	91.2% (0.763-0.981)	96.5% (0.926-1.000)	17.6% (0.120-0.232)	27.6%
TB-IGRA	79.1% (0.736-0.837)	88.6% (0.741-0.955)	98.0% (0.960-0.999)	37.8% (0.273-0.483)	67.7%
TB-DNA	45.6% (0.387-0.526)	100% (0.879-1.000)	100% (1.000-1.000)	21.1% (0.141-0.280)	45.6%
Xpert	72.4% (0.667-0.774)	100% (0.893-1.000)	100% (1.000-1.000)	30.5% (0.217-0.393)	72.4%
Culture	52.3% (0.465-0.581)	65.9% (0.511-0.781)	90.7% (0.862-0.952)	17.9% (0.120-0.238)	18.2%
AFS	10.7% (0.077-0.146)	95.7% (0.858-0.992)	94.3% (0.866-1.000)	14.0% (0.102-0.178)	6.4%
mNGS	90.1% (0.866-0.927)	75.0% (0.618-0.848)	96.3% (0.943-0.983)	51.3% (0.401-0.626)	65.1%
Parallel diagnostic test	100% (0.964-1.000)	31.3% (0.142-0.556)	90.4% (0.849-0.958)	100% (1.000-1.000)	31.3%
Serial diagnostic test	3.9% (0.015-0.096)	100% (0.806-1.000)	100% (1.000-1.000)	13.9% (0.076-0.202)	3.9%
Pulmonary samples					
TB-IgG	39.6% (0.327-0.468)	93.3% (0.787-0.988)	97.3% (0.936-1.000)	20.3% (0.136-0.270)	32.9%
TB-IGRA	78.9% (0.726-0.840)	87.1% (0.712-0.949)	97.5% (0.950-0.999)	39.7% (0.281-0.513)	66.0%
TB-DNA	47.2% (0.397-0.549)	100% (0.867-1.000)	100% (1.000-1.000)	22.5% (0.148-0.303)	47.2%
Xpert	77.3% (0.711-0.825)	100% (0.883-1.000)	100% (1.000-1.000)	38.2% (0.272-0.491)	77.3%
Culture	54.1% (0.475-0.605)	58.3% (0.422-0.729)	88.9% (0.836-0.942)	17.1% (0.104-0.237)	12.4%
AFS	12.9% (0.092-0.177)	97.5% (0.871-0.999)	96.9% (0.908-1.000)	15.7% (0.111-0.202)	10.4%
mNGS	93.4% (0.898-0.958)	81.0% (0.667-0.900)	96.9% (0.948-0.990)	65.4% (0.525-0.783)	74.4%
Parallel diagnostic test	100% (0.962-1.000)	33.3% (0.152-0.583)	90.6% (0.850-0.961)	100% (1.000-1.000)	33.3%
Serial diagnostic test	4.17% (0.016-0.102)	100% (0.796-1.000)	100% (1.000-1.000)	14.0% (0.074-0.206)	4.17%
Extrapulmonary samples					
TB-IgG	23.9% (0.116-0.362)	75.0% (0.326-1.000)	91.7% (0.760-1.000)	7.89%(-0.007-0.165)	-1.1%
TB-IGRA	80.0% (0.670-0.888)	100% (0.510-1.000)	100% (1.000-1.000)	28.6% (0.049-0.522)	80.0%
TB-DNA	36.7% (0.219-0.545)	100% (0.439-1.000)	100% (1.000-1.000)	13.6%(-0.007-0.280)	36.7%
Xpert	54.4% (0.416-0.666)	100% (0.439-1.000)	100% (1.000-1.000)	10.3%(-0.007-0.214)	54.4%
Culture	45.6% (0.334-0.584)	100% (0.676-1.000)	100% (1.000-1.000)	20.5% (0.078-0.332)	45.6%
AFS	2.9% (-0.011-0.070)	85.7% (0.598-1.000)	66.7% (0.133-1.000)	8.3% (0.020-0.147)	-11.4%
mNGS	81.4% (0.727-0.877)	50.0%(0.237-0.763)	94.3% (0.895-0.992)	20.8% (0.046-0.371)	31.4%
Parallel diagnostic test	100% (1.000-1.000)	0% (0-0)	87.5% (0.646-1.000)	NA	0%
Serial diagnostic test	0% (0-0)	100% (1.000-1.000)	NA	12.5%(-0.104-0.354)	0%

TB, tuberculosis; mNGS, metagenomic next-generation sequencing; IGRA, interferon-gamma release assays; TB-IgG, *Mycobacterium tuberculosis* IgG antibody; 95% CI, 95% confidence interval; PPV, positive predictive value; NPV, negative predictive value.

### 3.4 Diagnostic performance of TB-IgG, TB-IGRA, TB-DNA, Xpert, culture, AFS, and mNGS in TB

The diagnostic performance of TB-IgG, TB-IGRA, TB-DNA, Xpert, culture, AFS, mNGS, parallel diagnostic test, and serial diagnostic test was estimated according to different specimens (**Table 4**).

Among all clinically suspected TB specimens, mNGS reached a sensitivity of 90.1% (95% CI: 0.866–0.927), which was much higher than those of other traditional methods: culture (52.3%, 95% CI: 0.465–0.581), Xpert (72.4%, 95% CI: 0.667–0.774), TB-IGRA (79.1%, 95% CI: 0.736–0.837), TB-IgG (36.4%, 95% CI: 0.302–0.430), TB-DNA (45.6%, 95% CI: 0.387–0.526), and AFS (10.7%, 95% CI: 0.077–0.146). Conversely, the specificity of applying mNGS for detecting MTBC was 75% (95% CI: 0.618–0.848), which was slightly lower than those of Xpert (100%, 95% CI: 0.893–1.000), TB-DNA (100%, 95% CI: 0.879–1.000), TB-IgG (91.2%, 95% CI: 0.763–0.981), and TB-IGRA (88.6%, 95% CI: 0.741–0.955), but higher than that of traditional culture (65.9%, 95% CI: 0.511–0.781). Taking the clinical final diagnosis as the standard, the positive predictive value and negative predictive value of mNGS for MTBC detection were 96.3% and 51.3%, respectively. Compared with the other traditional methods, the results also showed that the Youden index of mNGS in all clinical samples was 65.1%, which was lower than those of Xpert (72.4%) and TB-IGRA (67.7%).

The diagnostic performance of mNGS and other methods in TB varied across different samples. In 313 pulmonary TB cases, mNGS identified 261 cases with a sensitivity of 93.4% (95% CI: 0.898–0.958), which was higher than those of the other conventional methods: culture (54.1%, 95% CI: 0.475–0.605), Xpert (77.3%, 95% CI: 0.711–0.825), TB-IGRA (78.9%, 95% CI: 0.726–0.840), TB-DNA (47.2%, 95% CI: 0.397–0.549), TB-IgG (39.6%, 95% CI: 0.327–0.468), and AFS (12.9%, 95% CI: 0.092–0.177). In 112 extrapulmonary TB cases, mNGS produced a sensitivity of 81.4% (95% CI: 0.727–0.877), which was higher than those of traditional methods: culture (45.6%, 95% CI: 0.334–0.584), Xpert (54.4%, 95% CI: 0.416–0.666), TB-IGRA (80.0%, 95% CI: 0.670–0.888), TB-DNA (36.7%, 95% CI: 0.219–0.545), TB-IgG (23.9%, 95% CI: 0.116–0.362), and AFS (2.9%, 95% CI: -0.011–0.070). The same specificity of TB-DNA and Xpert for detecting TB in 313 pulmonary samples from non-TB participants had been achieved (both 100%), while mNGS achieved only 81.0% (95% CI: 0.667–0.900). However, in 112 extrapulmonary samples from non-TB patients, the specificity of mNGS dropped to 50.0% (95% CI: 0.237–0.763), whereas traditional culture climbed to 100% (95% CI: 0.676–1.000). The Youden index of mNGS was calculated as 74.4% and 31.4% for pulmonary and extrapulmonary samples, respectively.

To better understand the diagnostic performance of TB-IgG, TB-IGRA, TB-DNA, Xpert, culture, AFS, and mNGS, we generated ROC curves and calculated the AUC. The AUC for mNGS (0.825, 95% CI: 0.754–0.897) was lower than that of the Xpert test (0.862, 95% CI: 0.819–0.905) and higher than those of the other tests (0.638, 95% CI: 0.551–0.725 for TB-IgG; 0.838, 95% CI: 0.770–0.907 for TB-IGRA; 0.728, 95% CI: 0.653–0.802 for TB-DNA; 0.591, 95% CI: 0.503–0.680 for culture; 0.532, 95% CI: 0.447–0.617 for AFS). These results suggested that mNGS had better diagnostic performance than the other clinical routine methods, except Xpert (**Figure 4**). In addition, analysis of the ROC curve for mNGS reads showed that the AUC for MTB diagnosis was 0.9073, with a sensitivity of 73.19% and specificity of 96.15% when the recommended cut-off value of 4 reads (Youden’s index 0.6934) was applied (**Figure 4B**).

**Figure 4:**
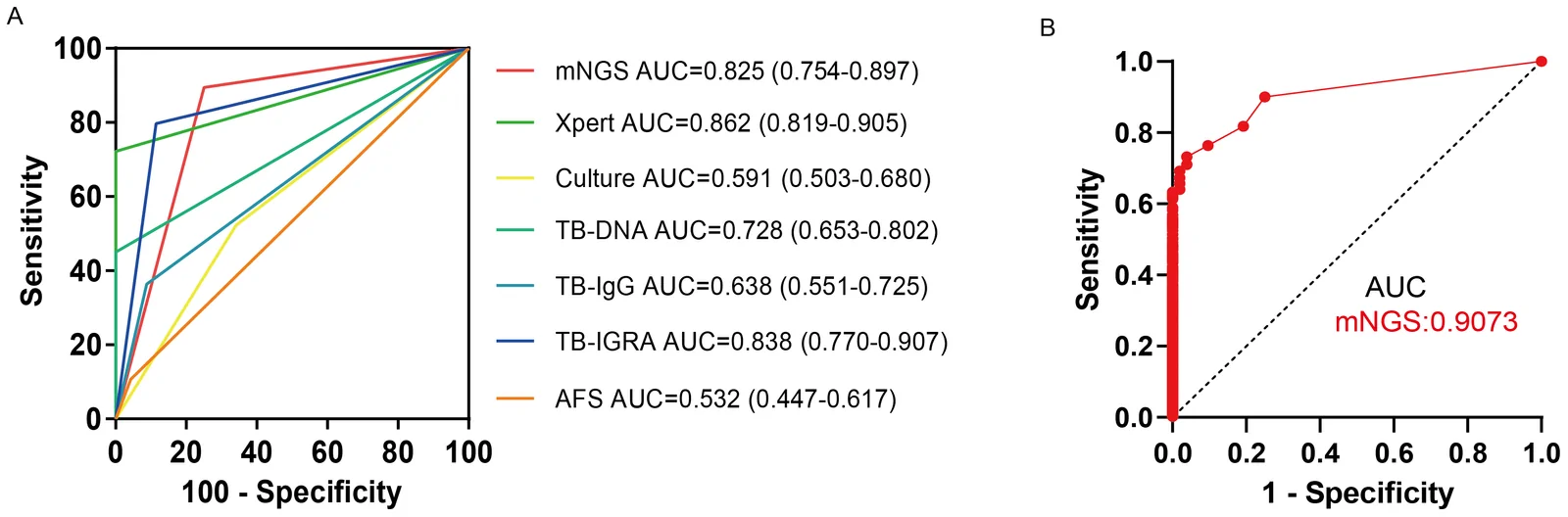
Diagnostic performance of all methods for distinguishing TB infection. **(A)** ROC curve of mNGS, TB-IgG, TB-IGRA, TB-DNA, Xpert, culture, and AFS individually for discrimination between TB infection and non-TB infection based on negative or positive results. **(B)** ROC curve of mNGS for discrimination between TB infection and non-TB infection based on read counts.

### 3.5 Diagnostic value of imaging for MTBC infection

To evaluate the value of imaging in diagnosing MTBC infection, we reviewed the imaging results of all cases and presented representative examples (**Figure 5**). Patient No.36 showed tuberculosis lesions in the lumbar vertebra and was diagnosed with lumbar tuberculosis. Patients No.10 and No.17 were both diagnosed with tuberculous meningoencephalitis; brain MRI imaging of these patients showed contrasting imaging findings, with normal and abnormal lesions, respectively. Patient No. 56, diagnosed with tuberculous pleurisy, displayed abnormal pleural lesions. Patient No. 2, diagnosed with tuberculous peritonitis, showed abnormal peritoneal lesions. Patient No. 34 exhibited abnormal renal lesions and was diagnosed with urinary tuberculosis. Patient No. 124, No. 104, and No. 40 all revealed abnormal pulmonary lesions, with or without co-infection with other pathogens. In this study, all diagnoses were based on expert clinical assessment according to patients’ clinical manifestations, clinical laboratory findings, and clinical imaging findings. Based on that, we found that imaging findings were typical for the diagnosis of lumbar TB but were difficult to interpret for TB involving other organs. Additionally, diagnosis was more challenging for radiologists in cases of co-infection with other pathogens. Therefore, it is of great significance for clinicians to consider clinical laboratory test results, including routine tests and mNGS, when making a diagnosis.

**Figure 5:**
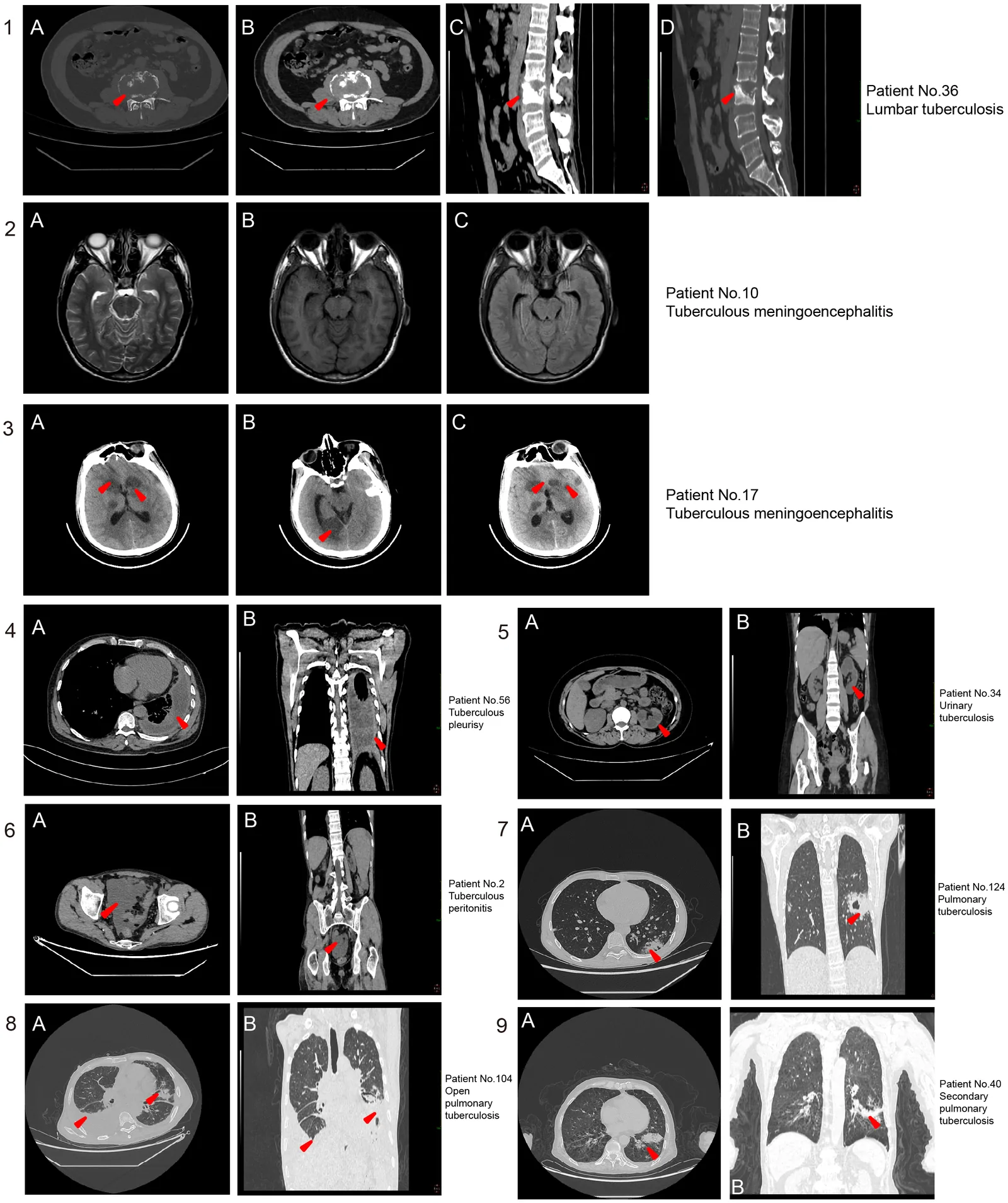
Representative imaging results of patients with lumbar tuberculosis, tuberculous meningoencephalitis, tuberculous pleurisy, urinary tuberculosis, tuberculous peritonitis, and pulmonary tuberculosis. For brain imaging, **(A)** refers to T1WI, **(B)** refers to T2WI, and **(C)** refers to fluid-attenuated inversion recovery. For chest imaging, the images include Tra/Axi and Sag.

### 3.6 Burden of MTBC affects the results of mNGS

The cell wall of MTBC is difficult to break, which makes DNA extraction for mNGS challenging. Hence, the sensitivity of MTBC detection by mNGS may be affected by the MTBC burden. Therefore, we compared MTBC read counts between patients with positive and negative results on other clinical routine tests. Of the 349 mNGS-positive samples, we found that the number of MTBC reads was significantly higher in TB-IgG-positive, TB-IGRA-positive, TB-DNA-positive, Xpert-positive, culture-positive, and AFS-positive groups (**Figures 6A–F**; **Supplementary Table 3**). As a result, positive findings on other clinical routine tests indicate a high MTBC burden. Therefore, our result indicates that MTBC burden affects the number of reads in mNGS. In our research, there were 35 mNGS-positive samples with only 1 read mapped to either the genus or species level of MTBC. Of these 35 samples, 15 cases were positive by Xpert and/or culture, and 10 cases (40%) were positive only by mNGS (**Figure 6G**). The remaining 10 cases were removed because they had no Xpert or culture results.

**Figure 6:**
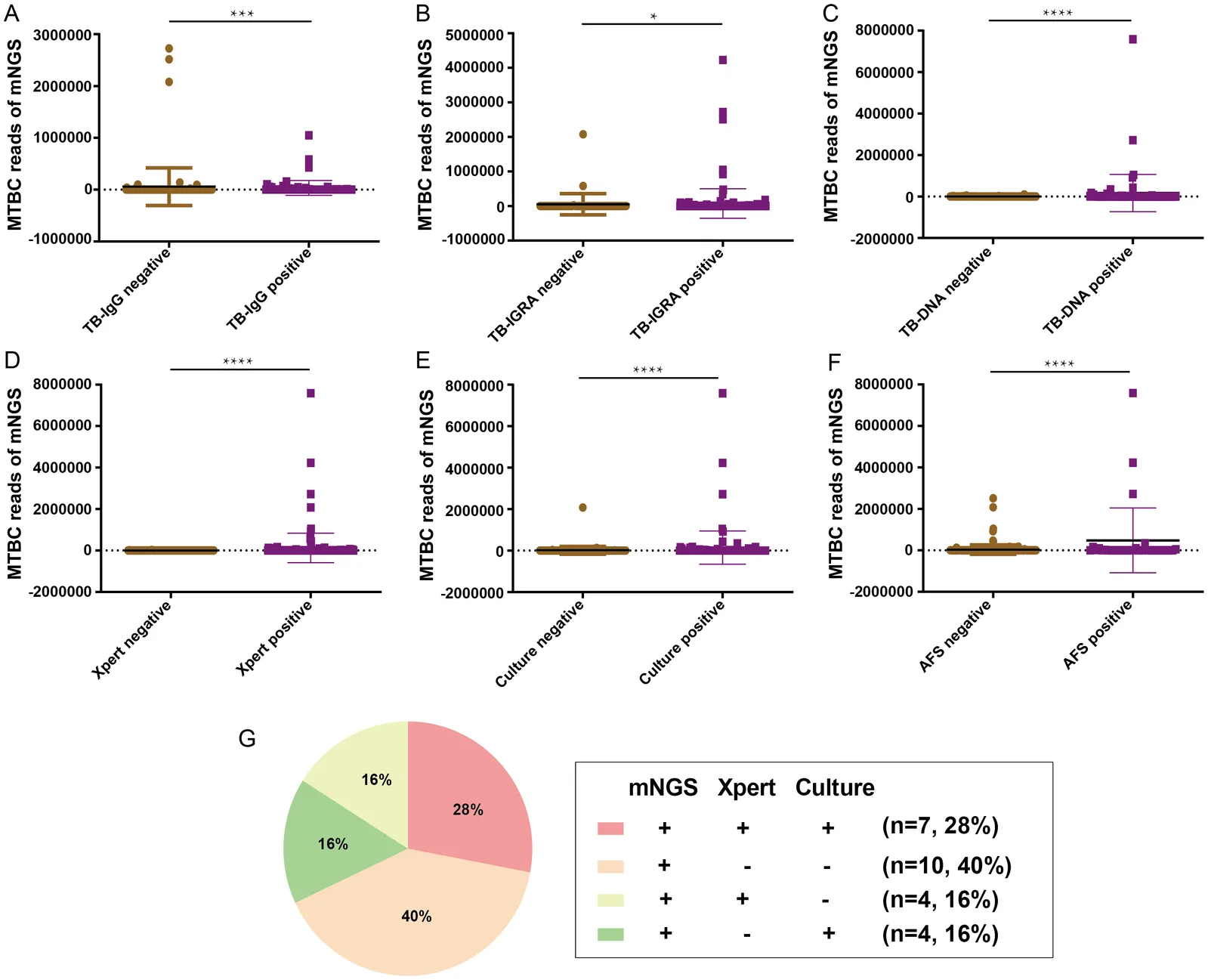
MTBC burden affects the number of MTBC reads detected by mNGS. **(A–F)** The number of reads is greater in positive samples than in negative samples according to the results of other routine clinical tests. **(G)** The detection results of mNGS, Xpert, and culture in patients with only 1 read mapping to either the genus or species level of MTBC by mNGS. *P < 0.05; ***P < 0.001, ****P < 0.0001..

### 3.7 Spearman correlation between IFN-γ release and lymphocytes in PTB, EPTB, and non-TB patients

IFN-γ release levels were positively correlated with lymphocyte counts (r = 0.2829, *P* < 0.0001) and lymphocyte percentages (r = 0.2927, *P* < 0.0001) in patients with PTB. Similarly, lymphocyte counts (r = 0.3982, *P* = 0.0397) and lymphocyte percentages (r = 0.6151, *P* = 0.0006) were positively correlated with IFN-γ concentrations in patients with EPTB (**Figure 7**). However, IFN-γ levels showed no correlation with lymphocyte counts and percentages in non-TB patients (*P* = 0.9115 and *P* = 0.7617, respectively). No correlations between IFN-γ release and mNGS reads were identified among the three groups (data not shown).

**Figure 7:**
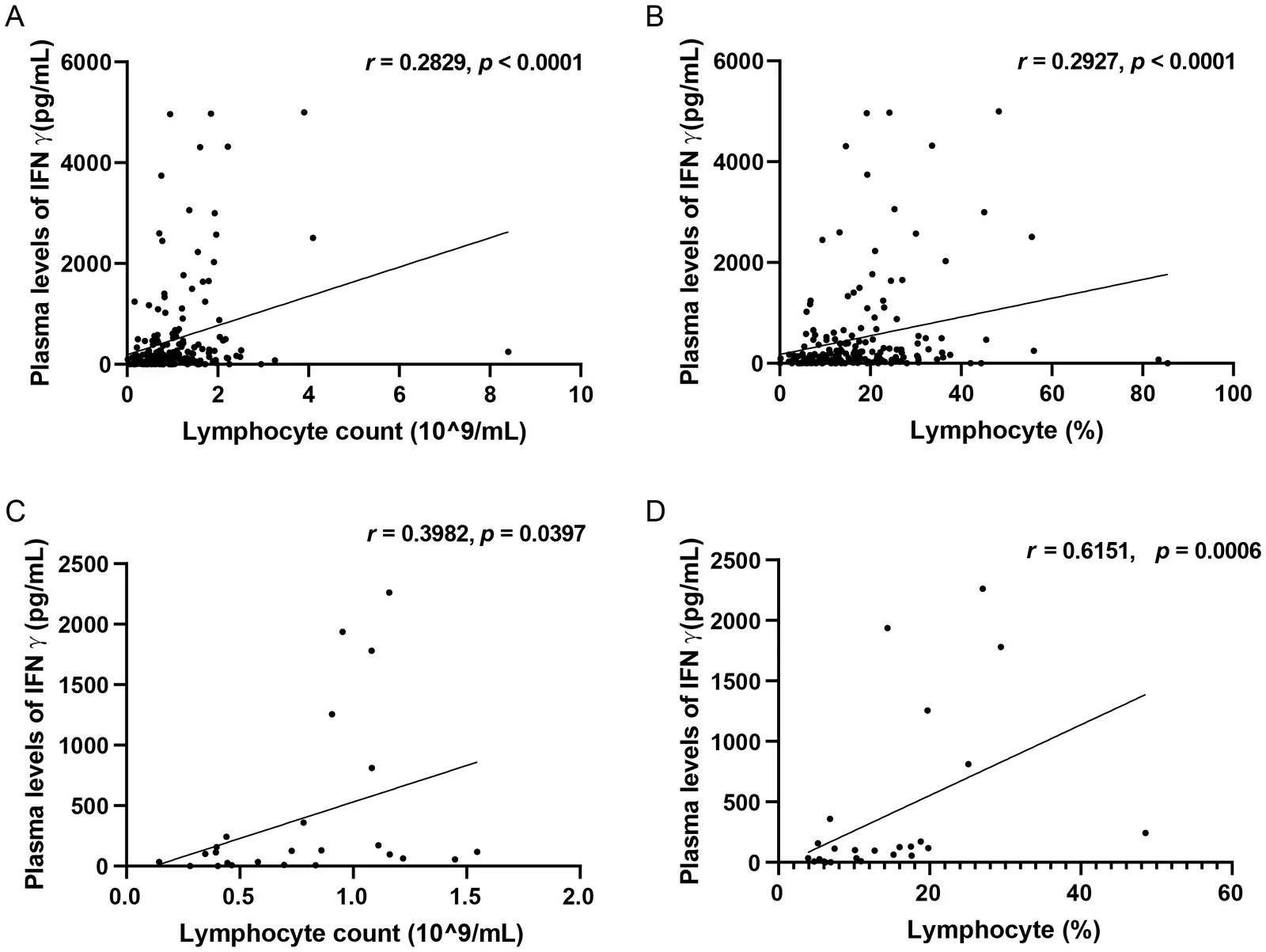
Analysis of correlations between plasma IFN-γ levels and lymphocyte counts and percentages in PTB and EPTB patients. **(A)** Correlation between plasma IFN-γ levels and lymphocyte counts in PTB patients. **(B)** Correlation between plasma IFN-γ levels and lymphocyte percentages in PTB patients. **(C)** Correlation between plasma IFN-γ levels and lymphocyte counts in EPTB patients. **(D)** Correlations between plasma IFN-γ levels and lymphocyte percentages in EPTB patients.

### 3.8 Infection characteristics and distribution of pathogens except MTBC

In our study, MTBC infection was defined primarily based on the clinician’s diagnosis, with consideration of microbial pathogens identified by either mNGS or culture methods, together with clinical symptoms and laboratory findings. MTBC infections were classified as either MTBC simple or MTBC mixed infections, with the latter accounting for approximately two-thirds of cases (**Figure 8B**). Except for MTBC infection, viruses (55.88% (171/306), 31.34% (21/67), and 59.62% (31/52) in the PTB, EPTB, and non-TB groups, respectively) represented the most common pathogens, followed by bacteria (37.25% (114/306), 7.46% (5/67), and 84.62% (44/52), respectively) and fungus (29.08% (89/306), 4.48% (3/67), and 48.08% (25/52), respectively) (**Figure 8A**). In addition to single infections, except MTBC, our results showed that the patients were infected by various combinations of pathogens, including viral, bacterial, and fungal coinfections; viral and bacterial co-infections; and bacterial and fungal coinfection (**Figure 8A**). Among the 55 microbes identified, the human gammaherpesvirus 4 (EBV) (n = 107/774) was the most commonly detected pathogen, followed by human betaherpesvirus 5 (CMV) (n = 79/774). The top five bacteria identified were *Enterococcus faecium* (n = 22), *Streptococcus pneumoniae* (n = 19), *Pseudomonas aeruginosa* (n = 18), *Acinetobacter baumannii* (n = 18), and *Klebsiella pneumoniae* (n = 16). *Candida albicans* was the most commonly detected fungus in patients with MTBC infection (**Figure 8C**). The pathogen species showed heterogeneous distributions across different sample types and groups. However, only a small number of pathogens were identified in CSF, whole blood, tissue, and body fluids, whereas BALF was associated with more pathogens (**Figure 8C**).

**Figure 8:**
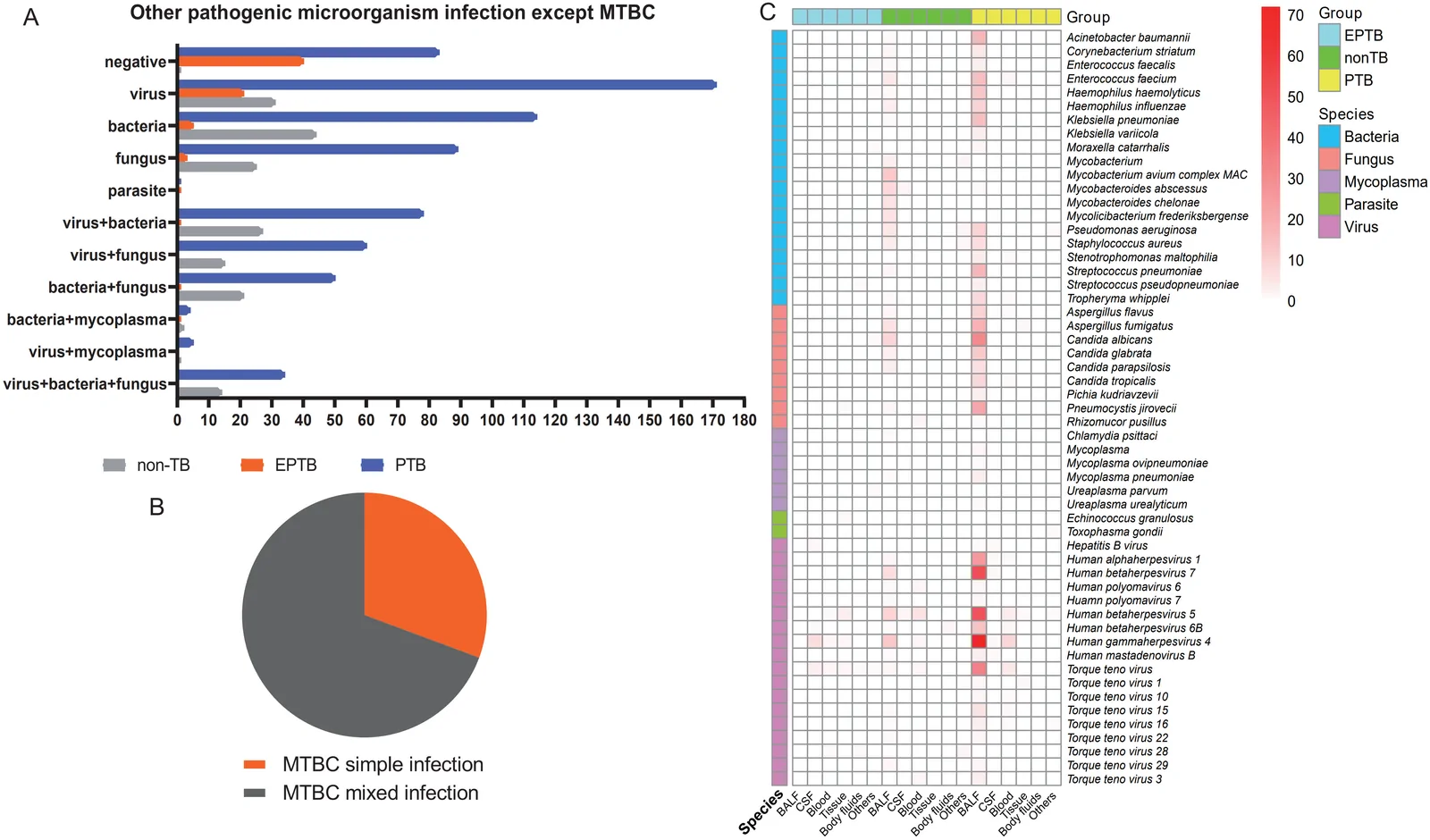
Types of infections and pathogen species other than MTBC. **(A)** Types of infection in the three groups: PTB, EPTB, and non-TB. **(B)** Proportions of MTBC simple and mixed infections. **(C)** The proportion of 55 pathogenic microorganisms in each sample type in the three groups.

### 3.9 Infection characteristics and analysis of NTM infection in the non-TB group

In this study, we notably found that NTM infections were most common in the non-TB group. Therefore, we analyzed the characteristics of NTM infections. In total, 40 cases were positive for NTM pathogens, with 39 in the non-TB group and 1 in the PTB group. This finding may be explained by our definition of MTBC infection, which was primarily based on the clinician’s diagnosis of TB infection according to clinical symptoms and other findings, including imaging, clinical manifestations, and laboratory results. Therefore, the one case with NTM infection in the PTB group was classified as PTB based on the diagnosis from the clinician. This finding may indicate that mNGS can potentially miss diagnosis because of the cell wall of MTBC. Among the 48 microbes identified, the top five NTM pathogens were *Mycobacterium avium complex MAC* (n = 12/128), *Mycobacteroides abscessus* (n = 12/128), *Mycobacterium* (n = 4/128), *Mycobacteroides chelonae* (n = 3/128), and *Mycolicibacterium frederiksbergense* (n = 3/128). The most commonly detected virus was human betaherpesvirus 5 (CMV) (n = 11/128). *Aspergillus fumigatus* was the most commonly detected fungus among patients with NTM-mNGS-positive results (**Figure 9A**). Notably, the pathogen species showed heterogeneous distributions across different sample types.

**Figure 9:**
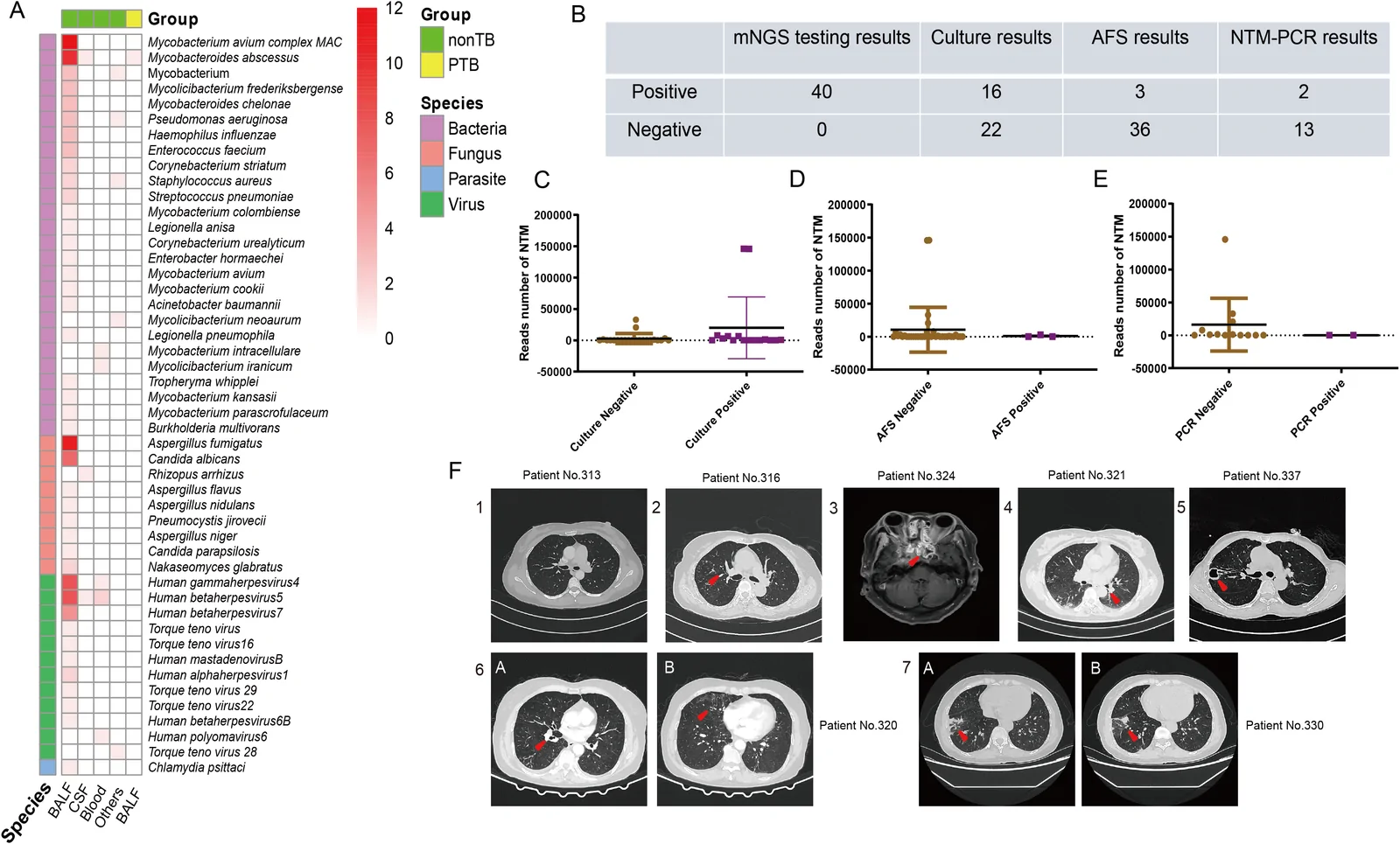
Representative imaging findings and mNGS results in NTM-mNGS-positive patients. **(A)** The top pathogenic microorganisms appeared in all 40 NTM-mNGS-positive samples. **(B)** Comparison of mNGS, conventional culture, AFS, and NTM-PCR. **(C–E)** NTM read counts in NTMPD patients is shown: the NTM read counts in the culture-positive group and the culture-negative group, AFS-positive group and AFS-negative group, and PCR-positive group and PCR-negative group. No significant differences were observed. **(F)** Representative imaging features of some NTM patients before treatment. The red arrowheads indicate abnormal lesions.

Additionally, we compared mNGS with other method for NTM detection, including culture, AFS, and NTM-PCR. The positive detection rate of mNGS was higher than those of culture, AFS, and NTM-PCR (**Figure 9B**). Among the 40 patients who were NTM-positive by mNGS, the sensitivity of mNGS for NTM detection was 100% (39/39) when compared with the final clinical diagnosis (39 NTM cases and 1 non-NTM case), and the positive predictive value was 97.5% (39/40). However, due to the limited number of negative cases (n = 1) and the absence of mNGS-negative results, specificity and negative predictive value could not be reliably assessed. Moreover, the small sample size may have led to an overestimation of the positive predictive value in this study. Furthermore, of the 40 NTM-mNGS-positive samples, we found that there was no correlation between the NTM read counts and the NTM-PCR-positive, culture-positive, and AFS-positive groups (**Figures 9C–E**), possibly because of the small sample size. We also reviewed all the imaging results of all NTM-mNGS-positive patients with a professional radiologist. Patient No. 313 showed no abnormal lesions in the lungs. Patient No. 316 showed solid nodules in the lung. Patient No. 320 showed small nodules, mainly distributed in the center of the bronchial lobules in the lungs. Patient No. 321 showed a tree-in-bud pattern in the lung. Patient No. 324 showed reversed halo sign in the brain. Patient No. 330 showed patchy consolidation, infiltrative consolidation, solid nodules, and peripheral GGO patchy exudation in the lung. Patient No. 337 showed bronchiectasis and cavities in the lung (**Figure 9F**).

## 4 Discussion

The aim of this study was to evaluate the clinical reliability and detection efficiency of mNGS diagnostic methods for distinguishing MTB from NTM in patients with all kinds of samples collected from pediatric to elderly patients. To this end, we compared mNGS results with those of commonly used routine methods and conducted a thorough chart review for each patient. Taking the clinicians’ diagnosis as the “gold standard”, we discovered that the sensitivity of mNGS diagnostic testing was 90.1%, similar to or higher than that reported for mNGS in other recent studies of suspected osteoarticular tuberculosis (Yan et al., 2024), disseminated tuberculosis (Ma et al., 2024), pulmonary tuberculosis (Shi et al., 2020b), and other forms of TB. The relatively high sensitivity of mNGS observed in our study warrants consideration. Several factors may have contributed to this estimate. First, we used clinicians’ final diagnosis as the gold standard, which may have resulted in a higher sensitivity estimate than that obtained using microbiological confirmation alone. Second, our inclusion criteria were restricted to patients with clinically suspected tuberculosis infection who presented with relevant symptoms and had imaging findings consistent with the diagnosis, which may have enriched the study population with a high pretest probability of disease. Finally, the relatively small sample size may have introduced statistical variability, potentially inflating the sensitivity estimate. Taken together, these factors may collectively explain the elevated sensitivity observed in our study. Conversely, the specificity was relatively lower than that of routine methods such as Xpert but higher than that of culture. More specifically, in the whole cohort, 13 patients in the non-TB group had potential MTB infection detected by mNGS with low reads per million (RPM) values, resulting in their classification as false positives in the final analysis, while negative results were obtained by culture, Xpert, and PCR in the same samples. This may be because this pathogen was not the point.

Although mNGS has emerged as a promising diagnostic tool for infectious diseases, its high sensitivity is often counterbalanced by relatively low specificity. The unbiased, broad-coverage, and high-throughput nature of mNGS enables rapid detection of rare or fastidious pathogens, in addition to the discovery of novel microorganisms (Chen et al., 2024; Zhao et al., 2024). However, this technology is associated with high costs and complex data interpretation. Its high sensitivity may increase the risk of false-positive results due to contamination by background microbiota or environmental microorganisms, while its suboptimal specificity could lead to the misidentification of non-pathogenic microorganisms as causative pathogens. Such misinterpretation might consequently trigger unnecessary anti-tuberculosis therapy and delay the identification of the true etiology (Chen et al., 2025; Wang et al., 2026). To mitigate these risks, a multidimensional clinical strategy should be implemented. First, positive mNGS results must be interpreted in the context of the patient’s clinical manifestations, radiological findings, and conventional laboratory tests. Second, for microorganisms suspected to represent contamination, confirmatory targeted assays (e.g., PCR) or dynamic monitoring are recommended. Third, given the variability in microbial composition across different clinical settings and specimen types, localized positive threshold criteria should be established to distinguish true pathogens from background microbiota (Chen et al., 2024; Wang et al., 2026; Zhao et al., 2024). The rational application of mNGS requires a delicate balance between sensitivity and specificity to avoid over-diagnosis and over-treatment.

In the past, mNGS tests were sent to centralized core facilities, as a result, the turnaround time (TAT) was approximately 3 to 4 days, which was a barrier for physicians when making a diagnosis (Wilson et al., 2019). However, in this study, TAT was reduced to 24–36 h owing to the sequencing platform being located within our hospital. Conversely, the average TAT of culture is 2 to 3 days for bacteria, 7 to 8 days for fungi, and 42 days for mycobacteria. Hence, mNGS tests have been suggested to serve as a familiar assistant method in clinical intractable cases when the pathogen must be identified as soon as possible, including *M. tuberculosis* infection (Lin et al., 2021; Zhu et al., 2021), *Treponema pallidum* infection (Liu et al., 2022; Zhou et al., 2022), *Leptospira santarosai* infection (Wilson et al., 2014), *Mycobacterium bovis* infection (Shi et al., 2020a), *Mycobacterium abscessus* infection (Xie et al., 2021), *Mycobacterium kansasii* infection (Shen et al., 2021), *Nocardia* infection (Jiao et al., 2022), anaerobic bacteria infection (Sun et al., 2024), and CNS infections or infections in immunocompetent individuals (Fourgeaud et al., 2024). Based on previous studies, we recommend that sample collection be performed from primary lesions in cases of suspected infection (Tao et al., 2022). Therefore, for MTB detection, mNGS can be used not only for pulmonary TB infection using BALF, sputum, and lung tissue samples (Dickson et al., 2014), but also for extrapulmonary TB infection using CSF (Wang et al., 2019; Yan et al., 2020), joint fluid (Mei et al., 2023), and abscesses (Zhao et al., 2020a). In this study, we collected multiple sample types, including ascitic fluid, pleural fluid, joint fluid, pus, tissues, secretions, sputum, urine, bone marrow, blood, BALF, and CSF. The positive rate of mNGS detection in all samples was 82.12%, compared with 90.52% in the PTB group, and 88.06% in the EPTB group, representing the highest positive rate among all methods.

In clinical practice, direct pathogen testing for *M. tuberculosis* is often used as the “gold standard” for diagnosing *M. tuberculosis* infection. However, due to the widespread use of the BCG vaccine in our country, this vaccination can affect the accuracy of tuberculosis infection diagnosis (Gao et al., 2015). TB culture is recognized as the “gold standard” because of its low cost and ability to support drug susceptibility testing; however, the test can take up to 8 weeks for visible colonies to grow on solid culture media and has low sensitivity and a low positive rate (Li et al., 2022b; Suárez et al., 2019). Acid-fast staining has the advantage of low cost and a short turnaround time, but its disadvantages include low sensitivity and a low positive rate. In addition, it is hard to distinguish between *Mycobacterium leprae* and NTM, and false-negative results are common in HIV-infected patients or children who bear low bacterial load (Figueredo et al., 2020; Getahun et al., 2007; Gopalaswamy et al., 2020; Li et al., 2022a). Similarly, in our study, TB culture and AFS showed sensitivities of 52.3% and 10.7%, respectively, in all samples; 54.1%, and 12.9%, respectively, in pulmonary samples; and 45.6% and 2.9%, respectively, in extrapulmonary samples (**Table 4**). These findings suggest that the MTB positive detection rate in extrapulmonary samples by microbiological methods was lower compared with pulmonary samples, which may be because of the difficulty of culturing or microscopically examining samples with a lower pathogen load in extrapulmonary disease.

Molecular testing methods, including TB-DNA and Xpert, have the advantages of short turnaround times, the ability to detect drug resistance, and high sensitivity and specificity, but their disadvantages include limited sensitivity in HIV-infected patients with miliary lung infiltrates, unimproved outcomes with enhanced testing, and the ability to detect only at specific sites (Dorman et al., 2018; Figueredo et al., 2020; Ho et al., 2016; Opota et al., 2019; Rudolf and Wejse, 2015). In our study, TB-DNA and Xpert showed sensitivities of 45.6% and 72.4%, respectively, in all samples; 47.2%, and 77.3%, respectively, in pulmonary samples; and 36.7% and 54.4%, respectively, in extrapulmonary samples (**Table 4**). In addition, the positive detection rates of TB-DNA and Xpert in all samples were 39.82% and 64.53%, respectively; those in the PTB group were 45.3% and 75.2%, respectively; and those in the EPTB group were both 50% (**Figure 3**). These findings suggest that Xpert showed not only better sensitivity but also better positive detection rate than TB-DNA. However, both the sensitivity and positive detection rate were lower than those of mNGS but higher than those of culture.

Immunological testing methods, including TB-IgG and TB-IGRA, are low-cost methods used for LTBI identification; howeverwhile, they have low sensitivity in immunocompromised populations and are unable to differentiate between LTBI and active TB (Anibarro and Pena, 2014; Auguste et al., 2017; Pai and Behr, 2016). The enzyme-linked immunosorbent assay (ELISA) principle has been adopted for TB-IGRA to detect human IFN in whole-blood samples stimulated with specific recombinant antigen of *Mycobacterium tuberculosis in vitro* (Sharma et al., 2017). TB-specific antigens are added to blood specimens or isolated peripheral monocytes from subjects, which are then incubated in culture. Upon re-encounter with specific *Mycobacterium tuberculosis* antigens, memory T cells from previously infected individuals can proliferate, differentiate, and release IFN-γ and other cytokines. The level of IFN-γ can then be quantified by ELISA to determine the presence of TB. Serial TB-IGRA testing is a dynamic test, and TB spot-forming cells may decrease over time if contacts are not continuously exposed to TB antigens (Zhang et al., 2019). Therefore, TB-IGRA false-positive results may occur in patients with a history of TB infection (Nenadić et al., 2012; Zhao et al., 2020b). However, there are conflicting data regarding the extent to which immunosuppression alters IGRA response. Some studies have shown that positive IGRA results are associated with higher CD4 cell counts, suggesting that the test may be compromised by immunosuppression (Jonnalagadda et al., 2010; Talati et al., 2009). Conversely, other studies have shown that tuberculosis IGRA responses are independent of CD4 cell counts (Leidl et al., 2010; Stephan et al., 2008). IGRA has some limitations in distinguishing active TB infection from latent TB infection, but it is useful for differentiating TB from other diseases. In our study, only 18 patients had a history of TB infection and positive TB-IGRA results. After TB infection, IFN-γ release levels were positively correlated with lymphocyte counts and lymphocyte percentages. This finding suggests that host immune status is closely related to TB-IgG and TB-IGRA results.

Imaging examinations can assist in diagnosis and follow-up but may be atypical in cases of co-infection or low immune status and are not specific for extrapulmonary TB (Rodriguez-Takeuchi et al., 2019; Skoura et al., 2015). Radiologists may be the first to distinguish MTB from NTM infection in patients who are asymptomatic or have non-specific constitutional symptoms, potentially leading to earlier diagnosis and management. In our study, imaging findings of lumbar tuberculosis may have been the easiest to identify. Patient No. 36 had simple TB infection in the lumbar region. Patient No. 10 showed no abnormal lesions in imaging, with 71 reads by mNGS. Conversely, patient No. 17 displayed simple TB infection in the brain. Patient No. 56 demonstrated simple infection, whereas the causative pathogen could not be determined in patient No. 2. Patient No. 34 showed simple infection, although mNGS detected bacterial and fungal co-infection. Patients No. 104 and 40 had TB co-infection with other inflammatory conditions involving bacteria, viruses, and fungi, as detected by mNGS. Non-tuberculous mycobacteria, which are ubiquitous environmental organisms that can be isolated from soil, water, fish, birds, and animals, can colonize the respiratory system without causing disease (Erasmus et al., 1999). NTM infections are usually slowly progressive, with imaging findings that may wax and wane over time. Patient No. 330 showed ground-glass shadow with suspected Aspergillus infection, which was not considered tuberculosis but rather NTM infection. Patient No. 337 displayed a hollow nodule, which indicated other causes in the background of bronchiectasis, which was suggestive of NTM infection according to the RSNA conference. Patient No. 324 demonstrated a reversed halo sign, but this finding could not indicate NTM infection. Altogether, imaging can serve as a direct diagnostic method for physicians only in some cases.

Despite being meticulously designed and analyzed, our study has some limitations. First, not all patients underwent all clinical tests due to the retrospective design of this study. Furthermore, among those who completed all tests, the limited specimen volume may have introduced random variability attributable to the small sample size. Second, our study is a single-center study with a relatively small sample size; particularly the number of samples from aseptic body fluid specimens, which may have introduced bias in the analysis results. Third, the clinical benefits, such as the impact of mNGS testing on length of hospital stay and patient prognosis compared with patients who did not undergo mNGS testing, require further investigation. Finally, which is of breakthrough significance, further research is needed to distinguish active MTB infection from latent MTB infection based on mNGS detection.

## 5 Conclusion

In conclusion, this study supports the potential role of mNGS as an adjunctive diagnostic approach in the management of suspected mycobacterial infections. Our data indicate that mNGS may offer greater sensitivity than routine methods for MTB detection and differentiation from NTM, thereby potentially expediting the diagnosis of these often indolent pathogens. Furthermore, its capacity to uncover polymicrobial co-infections in a single assay adds another dimension to its clinical utility. In challenging cases—regardless of patient age or specimen type—where conventional tests or imaging studies prove unrevealing, mNGS could provide clinically meaningful supplementary evidence to guide therapeutic decision-making. Nevertheless, we emphasize that mNGS should be viewed as a complement to, rather than a replacement for, conventional diagnostic methods.

## Data Availability

The datasets presented in this article are not readily available because of patient privacy concerns. Requests to access the datasets should be directed to the corresponding author.
