# Supplementary material for: Diagnostic performance of metagenomic next-generation sequencing in Mycobacterium and non-Mycobacterium infection patients: a retrospective study at a large comprehensive medical center
Source: Front Cell Infect Microbiol. 2026 Oct 29;16:1935536. doi: 10.3389/fcimb.2026.1935536 (PMC13754751; doi:10.3389/fcimb.2026.1935536)
Supplement: Supplementary file 1 [file Table1.docx]

**Table S1 MTB detection rate distribution in EPTB (n, %)**

|  | TB-IgG | TB-IGRA | TB-DNA | Xpert | Culture | AFS | mNGS |
| --- | --- | --- | --- | --- | --- | --- | --- |
| Tuberculosis meningitis/meningoencephalitis (n=24) | 0 | 10,41.67% | 3,12.5% | 12,50% | 12,50% | 0 | 20,83.33% |
| Tuberculous lymphadenitis (n=1) | 0 | 1,100% | - | - | - | - | 1,100% |
| Tuberculosis pleurisy (n=4) | 1,25% | 3,75% | - | 0 | 0 | 0 | 4,100% |
| Osteoarticular/Spinal/Joint tuberculosis (n=13) | 4,30.77% | 2,15.38% | - | 1,7.69% | 1,7.69% | 0 | 9,69.23% |
| Tuberculosis peritonitis (n=3) | 1,33.3% | 2,66.67% | 0 | 0 | 0 | 0 | 3,100% |
| Genitourinary tuberculosis (n=1) | 1,100% | 1,100% | 1,100% | - | - | - | 1,100% |
| Lumbar tuberculosis(n=17) | 4,23.53% | 3,17.65% | 2,11.76% | 1,5.89% | 2,11.76% | 1,5.89% | 14,82.35% |
| Skin tuberculosis(n=2) | - | - | - | 1,50% | 0 | 0 | 2,100% |

“-”refers to the tests are all NA.

**Table S2 Comparison of mNGS and conventional tests for *Mycobacterium* detection in PTB group**

| Method | Positive (n) | Negative (n) | Positive rate (%) |
| --- | --- | --- | --- |
| mNGS | 91 | 10 | 90.10 |
| Xpert | 78 | 23 | 77.23 |
| Culture | 63 | 38 | 62.38 |
| AFS | 13 | 88 | 12.87 |
| TB-IgG | 40 | 61 | 39.60 |
| TB-IGRA | 84 | 17 | 83.17 |
| TB-DNA | 45 | 56 | 44.55 |

**Table S3 Detailed statistics information of figure 6**

|  | Passed normality test | Passed variances (F) | Used test | P value (Two-tailed) |
| --- | --- | --- | --- | --- |
| TB-IgG | No | No | Mann-Whitney tset | 0.0003* |
| TB-IGRA | No | No | Mann-Whitney tset | 0.0291* |
| TB-DNA | No | No | Mann-Whitney tset | <0.0001* |
| Xpert | No | No | Mann-Whitney tset | <0.0001* |
| Culture | No | No | Mann-Whitney tset | <0.0001* |
| AFS | No | No | Mann-Whitney tset | <0.0001* |
